# The distribution of hypertrophy in anderson fabry disease

**DOI:** 10.1186/1532-429X-13-S1-P278

**Published:** 2011-02-02

**Authors:** Daniel M Sado, Sanjay M Banypersad, Perry M Elliott, James C Moon

**Affiliations:** 1 The Heart Hospital, London, UK

## Objective

To investigate the pattern of left ventricular hypertrophy (LVH) in Anderson Fabry Disease (AFD) using CMR.

## Background

AFD is an X linked disorder of lysosomal storage that can have various cardiac manifestations, most commonly resulting in LVH. Echocardiographic data on LVH distribution emphasised concentric hypertrophy in males and later onset in females with less hypertrophy. However this modality is often limited in visualisation of the left ventricular apex.

## Methods

27 patients (mean age 47, 33% males, a large cohort of female heterozygotes) with genetically proven AFD were assessed by CMR for hypertrophy, its distribution and LGE.

## Results

**Figure 1:**
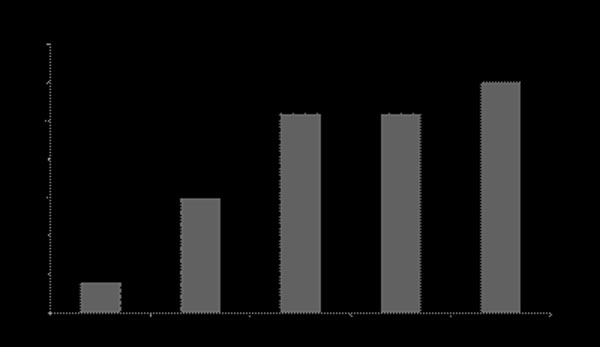
Distribution of hypertrophy in AFD

8 (30%) of patients had no LVH (Fig 1). Of those who did have LVH, 4 (21%) manifested an apical only distribution with 7 (37%) having Concentric Hypertrophy with Apical Cavity Obliteration (CHACO), 7 (37%) concentric hypertrophy with no apical cavity obliteration and 1 (5%) asymmetrical septal hypertrophy (ASH).

Echocardiography did not detect 4 (15%) of the patients (3 female) subsequently found to have hypertrophy on CMR, 1 (3%) of whom had an apical pattern.

LGE was seen in 12 (44%) of patients. Of these 12 patients, 6 (50%) had basal infero-lateral LGE only, with 3 (25%) having basal infero-lateral and other varying LGE distribution. A further 3 (25%) of patients had LGE only distributed away from the basal infero-lateral wall.

Of the 4 patients with apical hypertrophy, 2 (50%) had basal infero-lateral wall LGE, with 1 (25%) having apical LGE and the other no LGE.

## Conclusions

This study shows that AFD can manifest in an LV apical hypertrophy phenotype with most demonstrating apical cavity obliteration. It may be that this has not previously been recognised due to limitations of echocardiography in visualisation of the LV apex. Apical as well as basal infero-lateral LGE is also a manifestation of AFD.

